# A multi‐institutional evaluation of machine performance check system on treatment beam output and symmetry using statistical process control

**DOI:** 10.1002/acm2.12547

**Published:** 2019-02-20

**Authors:** Diana Binny, Trent Aland, Ben R. Archibald‐Heeren, Jamie V. Trapp, Tanya Kairn, Scott B. Crowe

**Affiliations:** ^1^ Icon Cancer Centres Northlakes QLD Australia; ^2^ Queensland University of Technology Brisbane QLD Australia; ^3^ Radiation Oncology Centres Wahroonga NSW Australia; ^4^ Cancer Care Services Royal Brisbane and Women's Hospital Brisbane QLD Australia

**Keywords:** EPID, machine performance check (MPC), quality assurance (QA), statistical process control (SPC)

## Abstract

**Background:**

The automated and integrated machine performance check (MPC) tool was verified against independent detectors to evaluate its beam uniformity and output detection abilities to consider it suitable for daily quality assurance (QA).

**Methods:**

Measurements were carried out on six linear accelerators (each located at six individual sites) using clinically available photon and electron energies for a period up to 12 months (n = 350). Daily constancy checks on beam symmetry and output were compared against independent devices such as the SNC Daily QA 3, PTW Farmer ionization chamber, and SNC field size QA phantom. MPC uniformity detection of beam symmetry adjustments was also assessed. Sensitivity of symmetry and output measurements were assessed using statistical process control (SPC) methods to derive tolerances for daily machine QA and baseline resets to account for drifts in output readings. I‐charts were used to evaluate systematic and nonsystematic trends to improve error detection capabilities based on calculated upper and lower control levels (UCL/LCL) derived using standard deviations from the mean dataset.

**Results:**

This study investigated the vendor's method of uniformity detection. Calculated mean uniformity variations were within ± 0.5% of Daily QA 3 vertical symmetry measurements. Mean MPC output variations were within ± 1.5% of Daily QA 3 and ±0.5% of Farmer ionization chamber detected variations. SPC calculated UCL values were a measure of change observed in the output detected for both MPC and Daily QA 3.

**Conclusions:**

Machine performance check was verified as a daily quality assurance tool to check machine output and symmetry while assessing against an independent detector on a weekly basis. MPC output detection can be improved by regular SPC‐based trend analysis to measure drifts in the inherent device and control systematic and random variations thereby increasing confidence in its capabilities as a QA device. A 3‐monthly MPC calibration assessment was recommended based on SPC capability and acceptability calculations.

## 1 INTRODUCTION

With increasing complexity in radiotherapy treatment delivery and automated treatment checks, quality assurance (QA) guidelines require significant updates to include evidence‐based tolerances for optimal machine performance. The primary aim of QA conceptually has involved ensuring that machine characteristics do not deviate from their baselines acquired during commissioning.^1^ Several national and international guidelines also recommend daily QA tests for radiotherapy treatment systems.^2^, ^3^, ^4^ Tests and tolerances in these guidelines, however, are based on traditionally adopted techniques to ensure an agreed upon standard of treatment quality is maintained.

The use of relative baseline comparisons of detector readings obtained during commissioning of a linear accelerator may not be sufficient or practical on a daily basis as QA checks are performed by treatment operators using cross‐calibrated detectors. These sophisticated and newly developed cross‐calibrated detector properties can vary significantly with radiation type, amount of exposure, dose rate, detector sensitivity, type of detector material, etc.^1^, ^5^ These cross‐calibrated detectors can be either independently purchased from a vendor^6^ or can be available as an integrated self‐check system within the treatment unit.^7^, ^8^ QA guidelines are clear about recommended tests for newer treatment techniques such as volumetric modulated arc therapy and stereotactic ablative/body radiotherapy SABR/SBRT instead there are no recommendations based on statistical process control (SPC) methods on the frequency of use and tolerance for the newly added and automated daily QA systems on linear accelerators.

Machine performance check (MPC) is one such integrated self‐check QA application released with the Varian TrueBeam 2.0 (Varian Medical Systems, Inc., Palo Alto, CA, USA) linear accelerator. The fully automated application uses the existing megavoltage (MV) electronic portal imaging device (EPID) and a kilovoltage (kV) on‐board imager (OBI) with and without the vendor supplied IsoCal^9^ ball bearing phantom to validate geometric and dosimetric capabilities of the treatment unit.^5^ OBI properties have been extensively evaluated by Yoo et al.^10^ while developing an OBI‐specific QA that tests safety and functionality, geometry, and image quality. EPIDs suffered from over response to low energy photons because its high atomic number increased the probability for photoelectric effect.^11^ In addition to this, the presence of backscattered radiation from the positioning arm itself affected its sensitivity by producing artifacts.^12^ The aS1200 amorphous silicon EPID released in version 2.0 TrueBeam Varian linear accelerator has advanced acquisition electronics and additional backscatter shielding resulting in improved dosimetric QA.^5^

Statistical methods^13^, ^14^, ^15^, ^16^, ^17^, ^18^ have been applied to independent and integrated radiotherapy QA systems to evaluate their functionality and recommend tolerances using existing knowledge from control charts and trend analysis. Several studies^16^, ^17^, ^19^, ^20^, ^21^ have also highlighted the importance of control charts in advanced radiotherapy. SPC^17^, ^18^, ^19^, ^20^, ^22^, ^23^, ^24^, ^25^, ^26^ is a quality control tool that applies control charts to a process to differentiate between systematic and unplanned behavior over time. Graphical techniques are applied to a process of interest to potentially improve the overall process by identifying random and nonrandom or planned drifts thereby deriving tolerances based on system performance and capabilities.

Clivio et al. and Barnes et al.^1^, ^27^ have explored MPC beam characteristics assessing its behavior against independent detectors and found this check system to be reliable and easy to use by comparing against independent detectors. However, there are currently no recommendations on MPC QA tolerances or frequency of baseline resets using SPC using long‐term multi‐institutional dataset for TrueBeam linear accelerators.

Apart from a study by Barnes et al. there are not many investigations carried out to test beam symmetry sensitivity to planned variations and quantify MPC tolerances to known errors. Variations in this study refer to relative baseline variations, that is, (measured value – baseline value)/baseline value × 100. In this work, we evaluate output and symmetry properties for photon and electron beams using statistical means for a period ranging from 4.5 months to a year.

## 2 METHODS

All measurements were carried out on six Varian TrueBeam v 2.0 linear accelerators (A–F). Linear accelerators B‐F were beam‐matched using beam quality indices TPR_20,10_ and R_50_ for photons and electrons, respectively, within ±0.5%. The beam quality index TPR_20,10_ (Photons) is the ratio of the absorbed dose in water at 20 and 10 cm depth, respectively, using a constant source‐chamber distance while R_50_ (electrons) refers to the half value depth in water at which the absorbed dose is 50% of its maximum value measured at a constant source‐surface distance. Dosimetric (output and symmetry) properties of all photon (6 and 10 MV) and electron (6, 9, 12, and 16 MeV) energies were assessed at a clinical maximum dose rate of 600 MU/min using devices listed in Table 1 for frequencies listed in Table 2.

**Table 1:** Measurement devices with assessed beam dimensions, their manufacturers, purpose, and nominal tolerances used during this study

Device (X × Y cm^2^)	Manufacturer	Purpose	Nominal Tolerances
Daily QA^™^ 3 (20 × 20 cm^2^)	Sun Nuclear Corporation, Melbourne, USA	Output, symmetry	±3%
30013 Farmer ionization chamber (IC) in Solid Water (10 × 10 cm^2^)	PTW, Freiburg, Germany	Output	±2%
SNC Machine^™^ FS‐QA (15 × 15 cm^2^)	Sun Nuclear Corporation, Melbourne, USA	Symmetry	±2%
MPC (13.3 × 13.3 cm^2^)	Varian Medical Systems, Palo Alto, CA, USA	Output, uniformity	±2%

**Table 2:** Routine QA frequencies and devices used during the analysis of this study

Machine	Frequency of tests				Analysis period (number of months)
	Daily QA 3	MPC	Number of measurements MPC/Daily QA 3	IC/SNC Machine	
A	Daily	Daily	98/98	Monthly	5
B	Daily	Daily	174/118	Monthly	4.5
C	Daily	Daily	119/97	Monthly	4.5
D	Daily	Daily	192/150	Monthly	7
E	Daily	Daily	265/224	Monthly	12
F	Once a week	Daily	190/87	Monthly	12

The Varian TrueBeam linear accelerator will be referred to as “TrueBeam” or “machine” in this study. Analysis in all cases were based on assessing variations from baseline values collected for each QA device during commissioning of the linear accelerator post absolute output measurements using the TRS‐398 protocol.^28^ Daily QA on each TrueBeam involved the use of MPC in conjunction with Daily QA 3 for linear accelerator dosimetric checks while SNC Machine Field Size (FS) QA phantom and Farmer ionization chamber (IC) in solid water baseline comparisons were conducted monthly as shown in Table 2. Photon and electron output measurements using the Farmer IC and solid water were made at reference FS mentioned in Table 1 at depths 5 and depth of maximum dose, respectively. Post monthly machine service, additional MPC measurements were made to ensure constancy in machine output and uniformity. This contributed to the higher number of MPC to daily QA 3 measurement ratio shown in Table 2. Twelve Farmer IC and SNC machine QA measurements from the 12‐month period were used in this study.

### 2.A Beam symmetry and uniformity

Daily QA 3 has been previously studied in great depth^15^ for the use of nominal tolerances for vertical and horizontal symmetry and output as stated in Table 1. Horizontal and vertical symmetry measurements are calculated in Daily QA 3 using eqs. (1) and (2), 15:

(1) $$\begin{array}{cc} & \mathrm{Horizontal}\;\;\;\mathrm{Symmetry}=\\ & \left({\left(-1\right)}^{1-\mathrm{OR}}.\left(\frac{T-B}{\mathrm{CAX}}\right).\left(1-\mathrm{AX}\right)+{\left(-1\right)}^{\mathrm{OR}}.\left(\frac{R-L}{\mathrm{CAX}}\right).\mathrm{AX}\right).100\% \end{array}$$

(2) $$\begin{array}{cc} & \mathrm{Vertical}\;\;\;\mathrm{Symmetry}=\\ & \left({\left(-1\right)}^{1-\mathrm{OR}}.\left(\frac{T-B}{\mathrm{CAX}}\right).\mathrm{AX}+{\left(-1\right)}^{\mathrm{OR}}.\left(\frac{R-L}{\mathrm{CAX}}\right).\left(1-\mathrm{AX}\right)\right).100\% \end{array}$$

Where the raw Top (T), bottom (B), left (L), right (R), and central axis (CAX) detector readings are used with the axial (AX) measurement value defined in the template for the detector axis such that: AX would be set to 0 for horizontal symmetry and 1 for vertical symmetry.^6^, ^15^ The orientation value of the measurement (OR) is set such that “Target” and “Right” = 1 and “Gun” and “Left” = 0.

IC QA for output and SNC Machine QA for symmetry tolerances, respectively, were conducted as per TG 142^2^ dosimetric and mechanical QA guidelines. To measure symmetry variations using the SNC Machine version 1.2.4 QA toolkit, a field size QA (FS‐QA) phantom was initially aligned to the cross‐hairs of the linear accelerator at gantry and collimator zero positions (G0H0). Following this, a mechanical visual check of various FSs was conducted using the field light and X and Y directional jaws on the treatment machine in conjunction with lines and markers on the FS‐QA phantom. A predetermined 15 × 15 cm^2^ FS at a 100 SSD (G0H0) was irradiated using 6 and 10 MV photon beams at a dose rate of 600 MU/min for 100 MU. Images collected at a predefined position using the EPID panel were automatically sent to the SNC Machine software for analysis via the integrated record and verify ARIA^®^ (Varian Medical Systems, Palo Alto, CA, USA) oncology information system. Horizontal and vertical jaw symmetry in SNC machine software were calculated using the normalized point difference method as shown in eq. (3).

(3) $$\mathrm{Symmetry}=\max\left(\frac{\left|L_{\mathrm{pos}}-R_{\mathrm{pos}}\right|}{P_{\mathrm{CAX}}}\right)\times 100$$

L_pos_ and R_pos_ are pixel values at positions equidistant from the CAX in the 80% FS region and P_CAX_ is the pixel value at the CAX. SNC machine was only used to assess photon beam symmetry, whereas Daily QA 3 was used to assess both photons and electron beam symmetry.

An MPC module was run daily using an 18 × 18 cm^2^ jaw defined FS at G0H0 positions. In‐order to reduce the impact of jaw positioning in the output and uniformity measurement, the beam characteristics are analyzed in the central 13.3 × 13.3 cm^2^ FS.^8^ According to the vendor, uniformity measurement variations in MPC depict a total percentage change in the central area of the imager after filtering the high‐frequency noise.^8^ The maximum variation between two imager pixels with the lowest and highest ratio gives a single result that comprised both vertical and horizontal planes with a potential of detecting symmetry variations.^8^, ^27^ Symmetry and uniformity variations from all detectors in this study were then input to a database to validate MPC uniformity measurements in detecting beam symmetry variations for planned and unplanned deviations. MPC and Daily QA 3 were subject to induced variations to quantify their sensitivity in detecting beam symmetry.

### 2.B Beam output

An MPC measured and analyzed beam output variation is a comparison between an average percentage variation detected in the central area of the imager to its corresponding baseline measurement. MPC evaluations heavily rely on an updated pixel map correction to avoid false QA failures. These corrections are performed monthly to cancel unwanted pixel values that accumulate as a result of overexposure or changed in sensitivity over time. Imaging calibrations that affect MPC include IsoCal calibration, MV imaging calibrations for high quality images and kV low dose.^8^ The MPC measured variations were compared against Daily QA 3 and monthly Farmer IC variation from baselines for all TrueBeams. Systematic changes in the machine beam output were detected by the Farmer IC and Daily QA3 devices over the assessment period. A corresponding shift in MPC output change was analyzed using SPC to statistically derive daily QA tolerances. The Farmer IC constancy was routinely evaluated using Strontium sources at each center and was also used to regulate drifts in MPC output variations. Daily QA 3 response is linked to the Farmer IC response such that a baseline reset is done if the variation between the two exceeds 1%. Box‐plot comparisons were made to visualize and assess relative mean MPC output variations against Daily QA3. The default value of whiskers in the boxplot corresponds to ±2.7*σ* and 99.3% coverage of the data as per the MATLAB program (The MathWorks, Natick, NA, USA).

### 2.C Statistical process control

Beam output information from all TrueBeams (A–F) were assessed using SPC to evaluate its sensitivity and uncertainty in the process and relay this information into treatment outcomes. Uncertainties can reside in a process in the form of a systematic or random behavior. In this study, control charts were used to impose upper and lower control limits (UCL and LCL) alongside a bold center line (CL) representing the average of the given dataset. UCL and LCL values were calculated in this study at ±3 standard deviations from the mean ($\overset{¯}{X}$) implying that 99.7% of the data points would fall within the control levels for a normally distributed dataset as shown in eqs. ((4), (5), (6)).

(4) $$\mathrm{UCL}=\bar{X}+3\frac{\bar{\mathrm{mR}}}{d_{2}\sqrt{n}}$$

(5) $$\mathrm{CL}=\bar{X}$$

(6) $$\mathrm{LCL}=\bar{X}-3\frac{\bar{\mathrm{mR}}}{d_{2}\sqrt{n}}$$

R is defined as the range of the group whereas d_2_ is a constant that depends on a continuous set of n measurements over a period. $\bar{\mathrm{mR}}$ is the absolute average of the moving range between two consecutive measurements and $\bar{X}$ is the mean of the dataset.^22^ In this study n is 1 as individual TrueBeams have been analyzed for the period they have remained active (as stated in Table 2), the constant d_2_ is 1.128.^29^ If all measurements fall within the upper and lower control levels, the process is said to be in control with random causes affecting the process. Out of process control behavior is indicated by measurement values residing outside the control levels and external influences such as investigating causes of the nonrandom behavior are then required to bring the process back into control.^25^ Random variations caused by human error due to mispositioning of the IsoCal phantom were immediately detected using the control chart method and were eliminated from the study after confirming with daily QA notes explaining the cause of repeat measurements. Normal distribution behavior for a dataset was assessed using the Anderson–Darling statistic using the below equation:

(7) $$A_{n}^{2}=-n-\sum_{i=1}^{n}\frac{2i-1}{n}\left[\ln\left(FX_{i}\right))+\ln\left(1-F\left(X_{n+1-i}\right)\right)\right]$$

Here a hypothesized distribution F(x) is evaluated for normality using ordered sample data points (X_1_ ˂ …. ˂ Xn) where n is the sample size for this data collected over time.^25^ The Anderson–Darling statistic was chosen to have a *α* — risk of 5% such that if $A_{n}^{2}$ is less than the *α*‐value, the data are normally distributed.

The process was also analyzed using capability (c_p_) and acceptability (c_pk_) ratios described in eqs. (8) and (9) to assess the system process for a nominal upper and lower specified level (USL and LSL set at ±3%). The ratios assess process behavior with respect to data spread within the specified levels (c_p_) and also assess if the data spread is close to the central value of the specifications (c_pk_) based on the standard deviation *σ* of a given distribution.^20^, ^25^

(8) $$c_{p}=\frac{\mathrm{USL}-\mathrm{LSL}}{6\;\;\sigma }$$

(9) $$c_{\mathrm{pk}}=\min\left(\frac{\mathrm{USL}-\bar{X}}{3\;\sigma },\frac{\bar{X}-\mathrm{LSL}}{3\;\sigma }\right)$$

A $c_{p}$ and $c_{\mathrm{pk}}$value of 1 would indicate that the process is within specifications and evenly distributed about the center of the upper and lower specification. The assumption in this case is that the target output variation is to be within 0.0% of the baseline value collected during commissioning/annual QA. $c_{p}$ and $c_{\mathrm{pk}}$values provide an estimate of the potential process or how the process would perform in the absence of special causes. The MATLAB program was used to calculate normality, capability, and acceptability values from the measurement data.

Even though a normal distribution is desired for SPC calculations it cannot always be the case as the measurements were assessed in a retrospective manner which can contain an out of control behavior that may contribute to non‐normal behavior but still be within the nominal specification.

## 3 RESULTS

### 3.A Beam symmetry and uniformity

**Figure 1:**
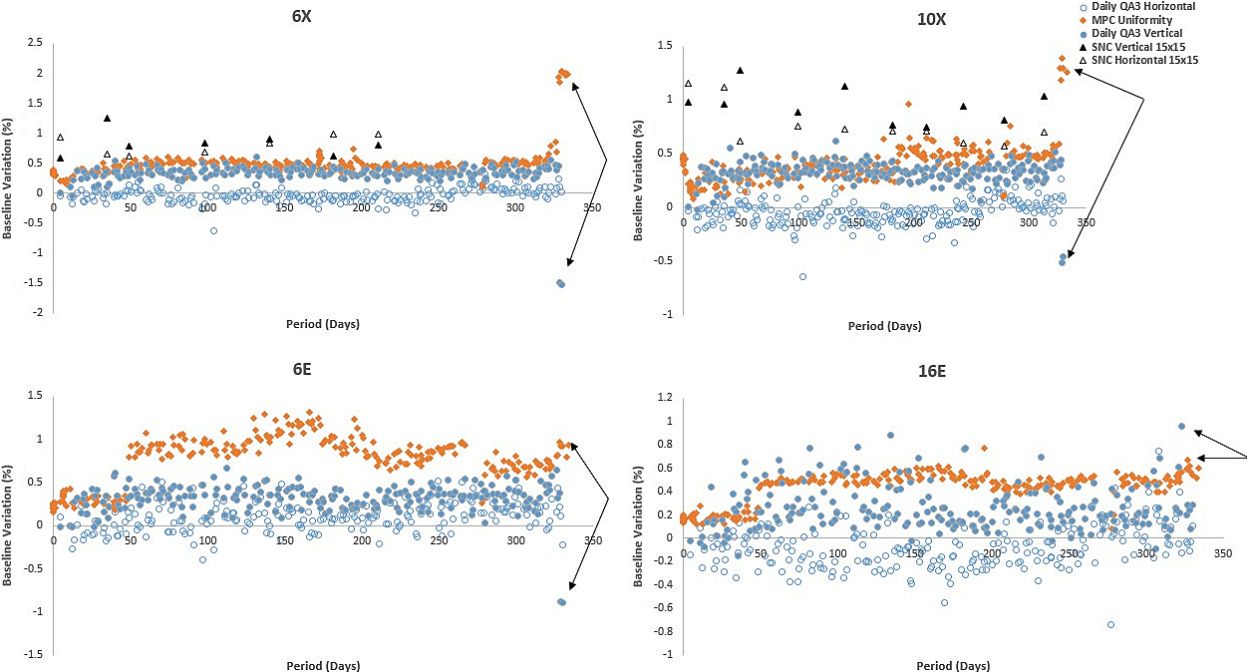
Baseline variation versus period: MPC, Daily QA 3 and SNC FS‐QA (photons only) symmetry and uniformity representation for machine E. Black arrows represent Daily QA 3 and MPC detection of induced beam symmetry adjustments.

Machine performance check daily uniformity variations from baselines were compared to symmetry variations measured using Daily QA 3 and SNC FS‐QA system devices. Using SPC analysis it was observed that MPC uniformity for all beam energies were within ±0.5% of the highest symmetry variations in vertical or horizontal direction. Machine E symmetry versus uniformity comparisons are shown in Fig. 1.

In the case of machine E, it was observed that vertical Daily QA 3 symmetry variations were within 0.4 ± 0.1% of its corresponding MPC uniformity. Beam uniformity for electrons was changed and SNC FS‐QA symmetry results were within ±1.5% of the corresponding Daily QA 3 results. A uniformity variation of ±0.5% was observed in the first 3 months of the TrueBeam being operational which was investigated using independent detectors: Daily QA 3 and SNC IC Profiler (Sun Nuclear Corporation, Melbourne, Vic., USA) and machine fault/service logs. This variation is in agreement with a previous MPC study by Barnes et al.^27^ No significant differences were observed from initial commissioning data and fault/service logs and the variation was assumed to be due to the inherent detection method used by MPC. MPC was observed to be sensitive to gradual output and symmetry changes over time (See Table 3).

**Table 3:** SPC‐Based Symmetry/Uniformity analysis “6X”

Machine	Symmetry/Uniformity (% variation) 6X					
		Data	UCL	LCL	$\overset{¯}{X}$	SD
A	MPC	98	0.44	0.22	0.33	0.15
	Daily QA3 H		0.27	−0.56	−0.14	0.25
	Daily QA3 V		0.42	−0.38	0.02	0.14
B	MPC	118	0.42	0.16	0.29	0.10
	Daily QA3 H		0.06	−0.34	−0.14	0.10
	Daily QA3 V		0.59	0.12	0.36	0.13
C	MPC	97	0.35	0.09	0.22	0.08
	Daily QA3 H		−0.02	−0.43	−0.23	0.09
	Daily QA3 V		0.25	−0.12	0.07	0.09
D	MPC	150	1.18	0.07	0.62	0.89
	Daily QA3 H		0.76	0.18	0.47	0.15
	Daily QA3 V		0.85	0.37	0.61	0.22
E	MPC	224	0.58	0.31	0.45	0.09
	Daily QA3 H		0.22	−0.28	−0.03	0.11
	Daily QA3 V		0.57	0.07	0.32	0.23
F	MPC	87	0.48	0.22	0.35	0.19
	Daily QA3 H		0.37	−0.24	0.06	0.21
	Daily QA3 V		1.08	0.59	0.83	0.10

Upper control limits calculated using SPC determined that MPC uniformity and daily QA3 vertical symmetry variations were within ±0.5% and mean variations calculated for all TrueBeams (See Table 4 and Tables S1–S5) also showed similar variations between the two devices. Machine D was subject to multiple MPC baseline resets due to service part replacements/output recalibrations carried out during this period which resulted in a higher SD of 0.89%. It was also observed that MPC and SNC FS‐QA measurements were within ±1% for photon beams (See Fig. 1).

**Table 4:** Mean variations between MPC uniformity and Daily QA3 vertical (V) and horizontal (H) symmetry calculated for TrueBeams A‐F for individual energies

Energy	MPC	DQA 3 V	DQA 3 H
6X	0.38	0.37	0.00
10X	0.33	0.26	−0.03
6E	0.50	0.29	0.10
9E	0.53	0.33	0.09
12E	0.50	0.29	0.06
16E	0.47	0.27	0.04

Inplane or vertical symmetry adjustments of 1.5% were made to photons (6 and 10 MV) and 0.8% were made to electron beams (6 and 16 MeV) and it was observed that MPC detected variations in photon and electron beams within ±0.5% of Daily QA 3 readings except in the case of 16E were detected variations were not significant (See Fig. 1 and Table 5).

**Table 5:** Relative absolute variations detected after planned vertical symmetry adjustments

Energy	Vertical symmetry (%) adjustment	DailyQA3 vertical symmetry change (%)	MPC uniformity change (%)
			
10X	1	0.9	1.2
6E	0.5	0.8	0.3
16E	0.5	0.2	0.1

Symmetry adjustments were detected by both MPC and Daily QA 3 devices with Daily QA 3 being more sensitive (within ±0.1%) to changes compared to MPC (within ±0.2%) for photon beams, whereas both systems respond within ±0.5% for electrons.

### 3.B Beam output

**Figure 2:**
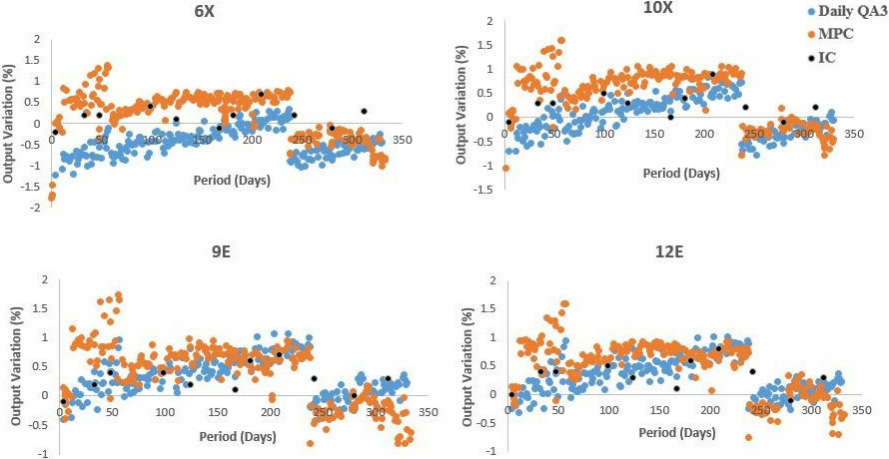
Output variations versus period: Farmer ionization chamber, Daily QA 3 and MPC output variations from baseline for Machine E.

Machine performance check output variations from baseline were plotted together with their corresponding Farmer IC and Daily QA 3 measurements as shown in Fig. 2.

**Figure 3:**
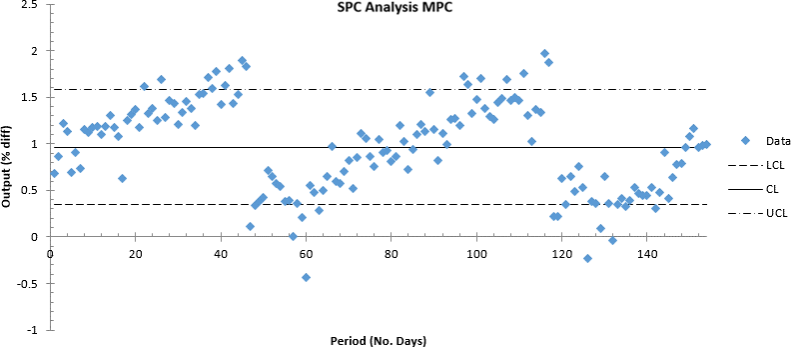
Output % variation versus period: MPC X‐control chart demonstrating UCL and LCL out of control measurements in a three‐monthly period for machine D.

**Figure 4:**
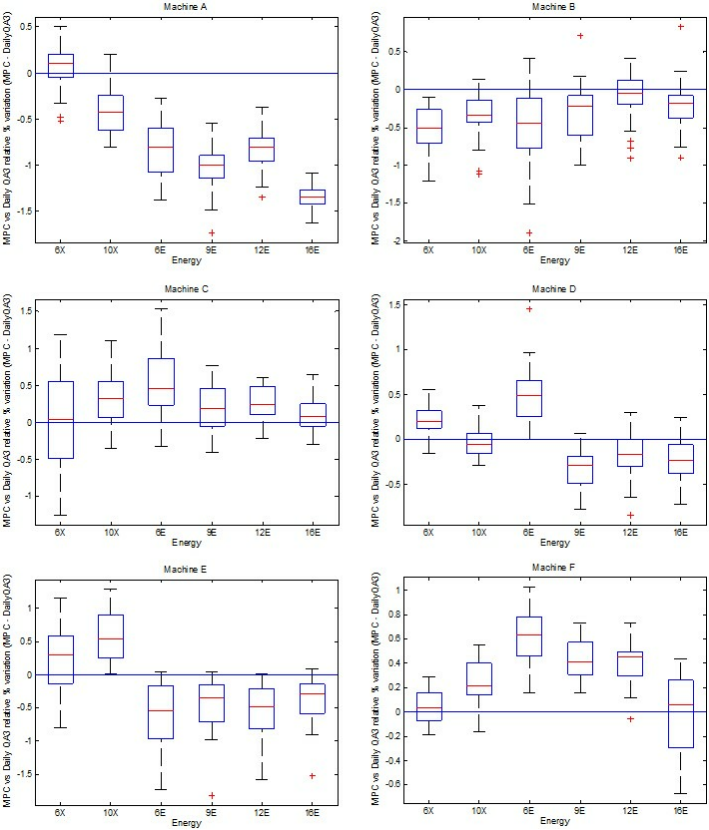
Relative MPC variations compared to Daily QA3 for all TrueBeams A–F.

Figures 2 and 3 show mean MPC output variations that were observed to be within ±0.5% of its corresponding Farmer IC variations and within ±1.5% when compared with Daily QA 3. On machine E, drifts were adjusted when the farmer IC reading exceeded or approached ±1% variation as shown in the period between 230 and 260 days in Fig. 2. MPC and Daily QA 3 resets were performed immediately after based on the Farmer IC baselines. MPC analysis showed UCL and LCL out of control measurements post a 3‐monthly period (See Figs. 2 and 3). As can be seen in the box plots in Fig. 4, it was observed that Machine A showed the highest relative mean MPC variation with respect to Daily QA 3 when compared to other linear accelerators (±1.5%) for electrons. TrueBeams B–F showed relative MPC output variations to be within ±0.5% of their corresponding Daily QA 3 readings for both photons and electrons.

Statistical process control mean analysis for all TrueBeams (A–F) showed that MPC and Daily QA 3 output variations were within ±1% for photons and electrons (except for machine A electrons). See Fig. 4, Table 6 and Tables S6 and S7 and Figure S1. Further investigations were carried into Machine A's electron variation measurements. It was noted that Daily QA 3 electron output calibrations were required to improve detection efficiency post machine output calibration and was recommended as a result of this analysis.

**Table 6:** SPC Analysis on MPC and Daily QA 3 output variations: “6X”

Machine	Output (% variation)					
	6X					
		Data	UCL	LCL	$\bar{X}$	SD
A	MPC	98	0.203	−0.745	−0.271	0.739
	Daily QA3	98	0.687	−0.610	0.038	0.613
B	MPC	174	0.727	−0.324	0.201	0.578
	Daily QA3	118	1.024	−0.028	0.498	0.534
C	MPC	119	0.388	−0.597	−0.104	0.383
	Daily QA3	97	1.127	−0.111	0.508	0.331
D	MPC	192	1.243	0.315	0.779	0.466
	Daily QA3	150	1.453	0.072	0.763	0.415
E	MPC	265	0.689	−0.254	0.218	0.539
	Daily QA3	224	0.053	−0.912	−0.430	0.343
F	MPC	190	0.286	−0.444	−0.079	0.292
	Daily QA3	87	0.881	−0.507	0.187	0.487

See UCL values for Table 6 and Tables S6 and S7 (Machine C and D).

Using SPC it was observed that higher UCL values corresponded to greater variations in the detection of machine output by both MPC and Daily QA 3.

**Figure 5:**
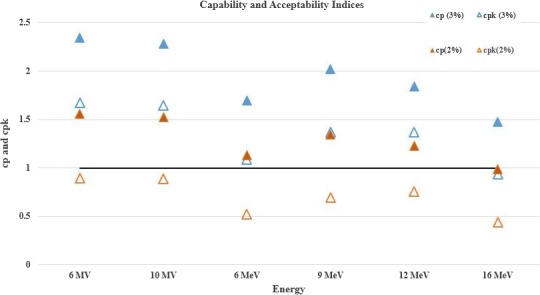
c_p_ and c_pk_ vs Energy: Three‐monthly capability and acceptability assessment to derive MPC machine output tolerance assessed for ±2% and ±3% USL and LSL and frequency of baseline calibration.

To derive machine tolerance for MPC output measurements, the Anderson–Darling test was run for a machine with the highest output variation after install and hence more probability of UCL/LCL out of control measurements (Machine D) to assess for normality following which capability and acceptability ratios were calculated. Since the nature of this assessment is retrospective, it does not allow for prospective changes to modify normality of a given dataset. It must be noted that normality is not a prerequisite for the assessment of capability ratios but is critical for its use in a process assessed in real‐time. In this study, we have simply noted that only 6 and 9 MeV data were normally distributed and yet assessed all variations against ±3% and ±2% specified levels on a 3‐monthly dataset. From Fig. 5, it was observed that all energies had their capability indices greater than 1 implying that the tolerance of ±2% is suitable, however, acceptability ratios suggested that the data dispersions were not normally distributed about its mean.

## 4 DISCUSSION

A multi‐center evaluation was performed on six TrueBeam linear accelerators to evaluate their MPC uniformity and output sensitivity against independent detectors like Daily QA 3, SNC FS‐QA system and Farmer IC. This study investigated the vendor's^27^ statement on uniformity detection where the total percentage change in the central area of the imager is used. From Table 4, it was observed that the total area change detected by MPC was in the same direction of the highest variation seen in Daily QA3. It was also observed that the reported mean uniformity variations for the TrueBeams were within ±0.5% of Daily QA 3 vertical symmetry variations. These results support findings presented by Barnes et al.^27^ however, is to be noted that this may not always be the case and each individual machine would require verifications to account for uncertainties in linear accelerator mechanical positions, set‐up uncertainties, detector sensitivity calibrations, focal spot positions, etc. Variations in horizontal and vertical directions can vary with MPC uniformity due to any of the previously mentioned reasons. Intentionally introduced symmetry variations of >0.5% were detected using MPC for all energies.

Mean MPC output variations were within ±0.5% of Farmer ICs and ±1.5% of Daily QA 3 detected variations. Output variations were found to be sensitive to frequency of Daily QA 3 detector calibrations as discussed by a previous publication.^15^ This drift was observed during analysis of Machine A. It was also noted that SPC calculated UCL values were a measure of change in the machine beam output detection parameters. This drift in MPC response has been reported in literature^27^ previously which may be due to gradual change in panel sensitivity. A 3‐monthly MPC assessment of baseline reset is recommended unless a beam output adjustment is performed earlier. MPC baseline resets must be performed post any beam output/symmetry adjustments and/or imager replacements. It is recommended that baseline resets on the MPC be linked to the departmental standard such that variations ≥2% from baseline trigger an investigation. Comparisons between the two detectors for output and uniformity checks and previous studies 1, 27 show agreement in calculated mean variations derived using SPC methods for photon and electron energies featured in this study. A future work for this study would be to make use of exponentially weighted moving average charts and actual process performance indices $P_{p}$ and $P_{\mathrm{pk}}$ to assess MPC measurement detection sensitivity by identifying slow drifts in the process. This would help tighten currently recommended tolerance levels when using control chart analysis in a prospective manner.

## 5 CONCLUSIONS

This study verified the capability of MPC output and uniformity detection for quality control on the TrueBeam linear accelerator daily. MPC Uniformity was found to be sensitive to symmetry variations greater than 0.5%. Confidence in daily MPC output detection can be improved by regular assessment on output drifts by comparing against an independent device such as Daily QA 3 on a weekly basis and a highly sensitive IC on a fortnightly to monthly basis. It is recommended that each machine MPC parameter be individually analyzed using SPC methods to derive tolerances specific to the machine to improve error detection capabilities and treatment efficiency.

## CONFLICT OF INTEREST

This research did not receive any specific grant from funding agencies in the public, commercial, or not‐for‐profit sectors.

## Supporting information


**Figure S1.** Individual MPC and Daily QA3 variation analysis for all machines (A–F).

**Table S1.** SPC‐Based Symmetry/Uniformity analysis 10 MV
**Table S2.** SPC‐Based Symmetry/Uniformity analysis 6 MeV
**Table S3.** SPC‐Based Symmetry/Uniformity analysis 9 MeV
**Table S4.** SPC‐Based Symmetry/Uniformity analysis 12 MeV
**Table S5.** SPC‐Based Symmetry/Uniformity analysis 16 MeV
**Table S6.** SPC‐Based Output Analysis: 10 MV
**Table S7.** SPC‐Based Output Analysis: 16 MeV
