# Supplementary figures and images for: A multi‐institutional evaluation of machine performance check system on treatment beam output and symmetry using statistical process control
Source: J Appl Clin Med Phys. 2019 Feb 20;20(3):71–80. doi: 10.1002/acm2.12547 (PMC6414149; doi:10.1002/acm2.12547)

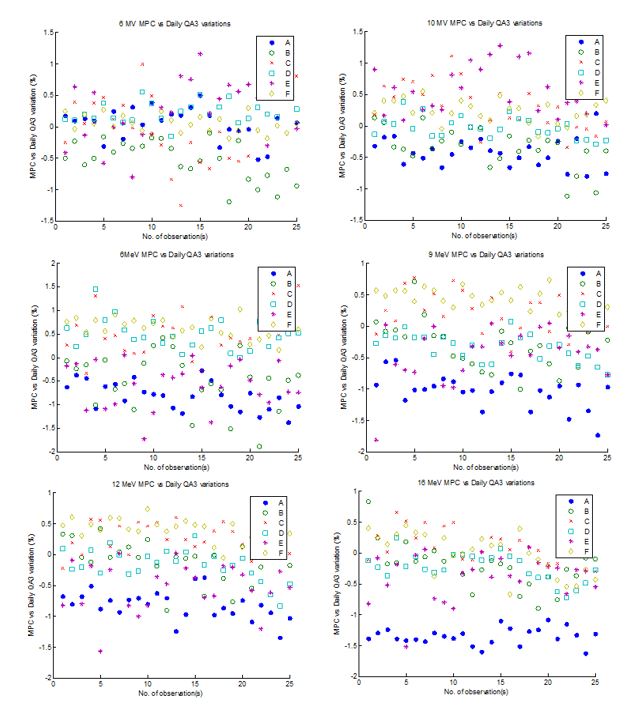

Supplement: Supplementary file 1 — Figure S1. Individual MPC and Daily QA3 variation analysis for all machines (A–F). [file ACM2-20-71-s001.jpg]
