# Supplementary material for: A multi‐institutional evaluation of machine performance check system on treatment beam output and symmetry using statistical process control
Source: J Appl Clin Med Phys. 2019 Feb 20;20(3):71–80. doi: 10.1002/acm2.12547 (PMC6414149; doi:10.1002/acm2.12547)
Supplement: Supplementary file 2 — Table S1. SPC‐Based Symmetry/Uniformity analysis 10 MV Table S2. SPC‐Based Symmetry/Uniformity analysis 6 MeV Table S3. SPC‐Based Symmetry/Uniformity analysis 9 MeV Table S4. SPC‐Based Symmetry/Uniformity analysis 12 MeV Table S5. SPC‐Based Symmetry/Uniformity analysis 16 MeV Table S6. SPC‐Based Output Analysis: 10 MV Table S7. SPC‐Based Output Analysis: 16 MeV [file ACM2-20-71-s002.docx]

List of Tables

Table 1. SPC based Symmetry/Uniformity analysis 10 MV

| Machine | Symmetry/Uniformity (% variation) 10 MV | | | | | |
| --- | --- | --- | --- | --- | --- | --- |
|  |  | Data | UCL | LCL | $\bar{X}$ | SD |
| A | MPC |  | 0.48 | 0.19 | 0.34 | 0.15 |
|  | Daily QA3 H | 100 | 0.29 | -0.71 | -0.21 | 0.24 |
|  | Daily QA3 V |  | 0.33 | -0.61 | -0.14 | 0.16 |
| B | MPC | 111 | 0.43 | 0.17 | 0.30 | 0.09 |
|  | Daily QA3 H |  | 0.06 | -0.39 | -0.16 | 0.11 |
|  | Daily QA3 V |  | 0.54 | 0.12 | 0.33 | 0.12 |
| C | MPC | 97 | 0.41 | 0.10 | 0.25 | 0.08 |
|  | Daily QA3 H |  | -0.01 | -0.43 | -0.22 | 0.09 |
|  | Daily QA3 V |  | 0.27 | -0.09 | 0.09 | 0.09 |
| D | MPC | 150 | 0.51 | 0.18 | 0.35 | 0.10 |
|  | Daily QA3 H |  | 0.80 | 0.22 | 0.51 | 0.15 |
|  | Daily QA3 V |  | 0.91 | 0.42 | 0.67 | 0.21 |
| E | MPC | 206 | 0.59 | 0.15 | 0.37 | 0.13 |
|  | Daily QA3 H |  | 0.22 | -0.31 | -0.05 | 0.12 |
|  | Daily QA3 V |  | 0.56 | 0.10 | 0.33 | 0.09 |
| F | MPC | 85 | 0.58 | 0.21 | 0.39 | 0.32 |
|  | Daily QA3 H |  | 0.29 | -0.34 | -0.02 | 0.22 |
|  | Daily QA3 V |  | 0.60 | 0.04 | 0.32 | 0.83 |

Table 2. SPC based Symmetry/Uniformity analysis 6 MeV

| Machine | Symmetry/Uniformity (% variation) 6 MeV | | | | | |
| --- | --- | --- | --- | --- | --- | --- |
|  |  | Data | UCL | LCL | $\bar{X}$ | SD |
| A | MPC | 99 | 0.71 | 0.10 | 0.41 | 0.16 |
|  | Daily QA3 H |  | 0.88 | -0.58 | 0.15 | 0.29 |
|  | Daily QA3 V |  | 0.80 | -0.51 | 0.14 | 0.22 |
| B | MPC | 111 | 0.51 | 0.20 | 0.36 | 0.08 |
|  | Daily QA3 H |  | 0.51 | -0.18 | 0.16 | 0.13 |
|  | Daily QA3 V |  | 0.79 | 0.07 | 0.43 | 0.18 |
| C | MPC | 99 | 0.86 | 0.17 | 0.52 | 0.16 |
|  | Daily QA3 H |  | 0.35 | -0.82 | -0.23 | 0.20 |
|  | Daily QA3 V |  | 0.86 | -0.50 | 0.18 | 0.28 |
| D | MPC | 150 | 0.86 | 0.31 | 0.59 | 0.20 |
|  | Daily QA3 H |  | 0.40 | -0.58 | -0.09 | 0.20 |
|  | Daily QA3 V |  | 0.93 | -0.23 | 0.35 | 0.26 |
| E | MPC | 224 | 0.99 | 0.51 | 0.75 | 0.31 |
|  | Daily QA3 H |  | 0.52 | -0.29 | 0.11 | 0.17 |
|  | Daily QA3 V |  | 0.67 | -0.06 | 0.31 | 0.19 |
| F | MPC | 87 | 0.68 | 0.15 | 0.41 | 0.09 |
|  | Daily QA3 H |  | 1.12 | -0.12 | 0.50 | 0.25 |
|  | Daily QA3 V |  | 0.83 | -0.15 | 0.34 | 0.20 |

Table 3. SPC based Symmetry/Uniformity analysis 9 MeV

| Machine | Symmetry/Uniformity (% variation) 9 MeV | | | | | |
| --- | --- | --- | --- | --- | --- | --- |
|  |  | Data | UCL | LCL | $\bar{X}$ | SD |
| A | MPC | 98 | 0.76 | 0.19 | 0.47 | 0.20 |
|  | Daily QA3 H |  | 0.49 | -0.51 | -0.01 | 0.22 |
|  | Daily QA3 V |  | 0.67 | -0.40 | 0.14 | 0.18 |
| B | MPC | 110 | 0.53 | 0.16 | 0.35 | 0.09 |
|  | Daily QA3 H |  | 0.43 | -0.02 | 0.21 | 0.09 |
|  | Daily QA3 V |  | 0.80 | 0.28 | 0.54 | 0.18 |
| C | MPC | 96 | 0.78 | 0.19 | 0.48 | 0.14 |
|  | Daily QA3 H |  | 0.11 | -0.50 | -0.19 | 0.13 |
|  | Daily QA3 V |  | 0.63 | -0.12 | 0.25 | 0.14 |
| D | MPC | 150 | 0.92 | 0.45 | 0.68 | 0.29 |
|  | Daily QA3 H |  | 0.43 | -0.25 | 0.09 | 0.16 |
|  | Daily QA3 V |  | 0.73 | 0.10 | 0.41 | 0.21 |
| E | MPC | 224 | 1.10 | 0.39 | 0.75 | 0.68 |
|  | Daily QA3 H |  | 0.30 | -0.28 | 0.01 | 0.12 |
|  | Daily QA3 V |  | 0.75 | 0.11 | 0.43 | 0.21 |
| F | MPC | 87 | 0.67 | 0.19 | 0.43 | 0.10 |
|  | Daily QA3 H |  | 0.78 | 0.11 | 0.45 | 0.17 |
|  | Daily QA3 V |  | 0.54 | -0.08 | 0.23 | 0.14 |

Table 4. SPC based Symmetry/Uniformity analysis 12 MeV

| Machine | Symmetry/Uniformity (% variation) 12 MeV | | | | | |
| --- | --- | --- | --- | --- | --- | --- |
|  |  | Data | UCL | LCL | $\bar{X}$ | SD(%) |
| A | MPC | 99 | 0.684 | 0.190 | 0.437 | 0.166 |
|  | Daily QA3 H |  | 0.541 | -0.539 | 0.001 | 0.292 |
|  | Daily QA3 V |  | 0.656 | -0.345 | 0.156 | 0.182 |
| B | MPC | 111 | 0.45 | 0.17 | 0.31 | 0.08 |
|  | Daily QA3 H |  | 0.43 | -0.05 | 0.19 | 0.10 |
|  | Daily QA3 V |  | 0.70 | 0.27 | 0.49 | 0.19 |
| C | MPC | 93 | 0.68 | 0.18 | 0.43 | 0.11 |
|  | Daily QA3 H |  | 0.20 | -0.39 | -0.10 | 0.12 |
|  | Daily QA3 V |  | 0.62 | 0.01 | 0.32 | 0.13 |
| D | MPC | 150 | 1.13 | 0.63 | 0.88 | 0.45 |
|  | Daily QA3 H |  | 0.54 | -0.20 | 0.17 | 0.22 |
|  | Daily QA3 V |  | 0.62 | 0.12 | 0.37 | 0.27 |
| E | MPC | 222 | 0.73 | 0.39 | 0.56 | 0.21 |
|  | Daily QA3 H |  | 0.32 | -0.31 | 0.01 | 0.14 |
|  | Daily QA3 V |  | 0.70 | 0.01 | 0.35 | 0.14 |
| F | MPC | 87 | 0.58 | 0.18 | 0.38 | 0.12 |
|  | Daily QA3 H |  | 0.38 | -0.19 | 0.09 | 0.20 |
|  | Daily QA3 V |  | 0.33 | -0.23 | 0.05 | 0.26 |

Table 5. SPC based Symmetry/Uniformity analysis 16 MeV

| Machine | Symmetry/Uniformity (% variation) 16 MeV | | | | | |
| --- | --- | --- | --- | --- | --- | --- |
|  |  | Data | UCL | LCL | $\bar{X}$ | SD |
| A | MPC | 87 | 0.62 | 0.21 | 0.42 | 0.16 |
|  | Daily QA3 H |  | 0.55 | -0.47 | 0.04 | 0.30 |
|  | Daily QA3 V |  | 0.53 | -0.31 | 0.11 | 0.18 |
| B | MPC | 110 | 0.41 | 0.18 | 0.30 | 0.08 |
|  | Daily QA3 H |  | 0.36 | -0.14 | 0.11 | 0.10 |
|  | Daily QA3 V |  | 0.71 | 0.28 | 0.50 | 0.19 |
| C | MPC | 95 | 0.55 | 0.17 | 0.36 | 0.09 |
|  | Daily QA3 H |  | 0.28 | -0.51 | -0.12 | 0.17 |
|  | Daily QA3 V |  | 0.61 | -0.01 | 0.30 | 0.16 |
| D | MPC | 142 | 1.21 | 0.80 | 1.00 | 0.59 |
|  | Daily QA3 H |  | 0.76 | -0.14 | 0.31 | 0.24 |
|  | Daily QA3 V |  | 0.68 | 0.23 | 0.45 | 0.31 |
| E | MPC | 202 | 0.53 | 0.30 | 0.42 | 0.15 |
|  | Daily QA3 H |  | 0.33 | -0.51 | -0.09 | 0.17 |
|  | Daily QA3 V |  | 0.69 | -0.19 | 0.25 | 0.19 |
| F | MPC | 87 | 0.47 | 0.13 | 0.30 | 0.12 |
|  | Daily QA3 H |  | 0.28 | -0.32 | -0.02 | 0.21 |
|  | Daily QA3 V |  | 0.24 | -0.21 | 0.02 | 0.28 |

Table 6. SPC Based Output Analysis: 10 MV

|  | Output (% variation) | | | | | |
| --- | --- | --- | --- | --- | --- | --- |
| Machine | **10 MV** | | | | | |
|  |  | Data | UCL | LCL | $\bar{X}$ | SD |
| A | MPC | 100 | 0.462 | -0.419 | 0.021 | 0.633 |
|  | Daily QA3 | 100 | 0.697 | -0.458 | 0.120 | 0.507 |
| B | MPC | 154 | 0.575 | -0.343 | 0.116 | 0.503 |
|  | Daily QA3 | 111 | 1.279 | 0.238 | 0.759 | 0.512 |
| C | MPC | 112 | 0.497 | -0.467 | 0.015 | 0.367 |
|  | Daily QA3 | 98 | 1.116 | -0.263 | 0.426 | 0.398 |
| D | MPC | 156 | 1.386 | 0.438 | 0.912 | 0.415 |
|  | Daily QA3 | 150 | 1.303 | 0.090 | 0.697 | 0.390 |
| E | MPC | 241 | 0.891 | -0.072 | 0.409 | 0.566 |
|  | Daily QA3 | 223 | 0.457 | -0.399 | 0.029 | 0.377 |
| F | MPC | 173 | -0.322 | 0.440 | 0.059 | 0.344 |
|  | Daily QA3 | 85 | 1.294 | -0.117 | 0.588 | 0.799 |

Table 7. SPC Based Output Analysis: 16 MeV

|  | Output (% variation) | | | | | |
| --- | --- | --- | --- | --- | --- | --- |
| Machine | **16 MeV** | | | | | |
|  |  | Data | UCL | LCL | $\bar{X}$ | SD |
| A | MPC | 87 | 0.677 | -0.134 | 0.271 | 0.238 |
|  | Daily QA3 | 87 | 1.209 | -0.102 | 0.553 | 0.437 |
| B | MPC | 144 | -0.246 | -1.134 | -0.690 | 0.604 |
|  | Daily QA3 | 110 | 1.157 | -0.055 | 0.551 | 0.505 |
| C | MPC | 108 | 0.471 | -0.353 | 0.059 | 0.416 |
|  | Daily QA3 | 94 | 1.060 | -0.386 | 0.337 | 0.497 |
| D | MPC | 155 | 1.094 | 0.180 | 0.637 | 0.773 |
|  | Daily QA3 | 140 | 0.888 | -0.354 | 0.267 | 0.699 |
| E | MPC | 234 | 0.820 | 0.028 | 0.424 | 0.451 |
|  | Daily QA3 | 223 | 0.761 | -0.139 | 0.311 | 0.338 |
| F | MPC | 171 | 0.278 | -0.357 | -0.039 | 0.249 |
|  | Daily QA3 | 87 | 1.609 | 0.218 | 0.913 | 0.488 |


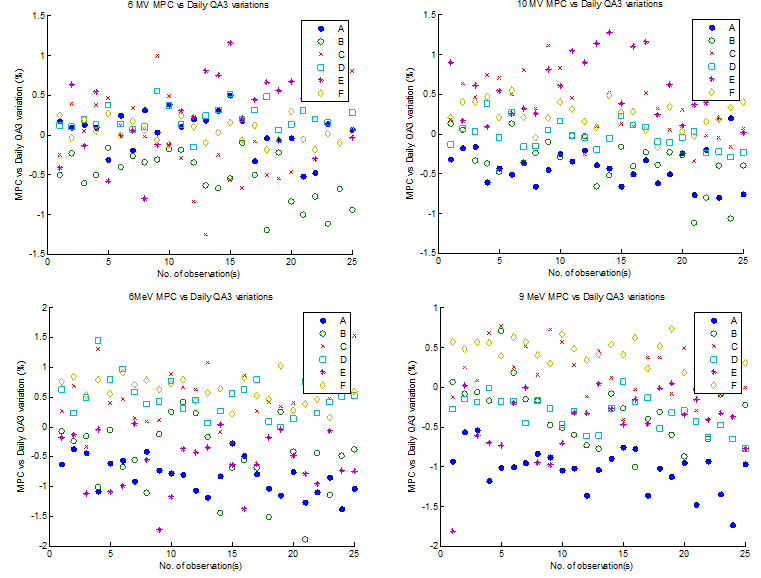

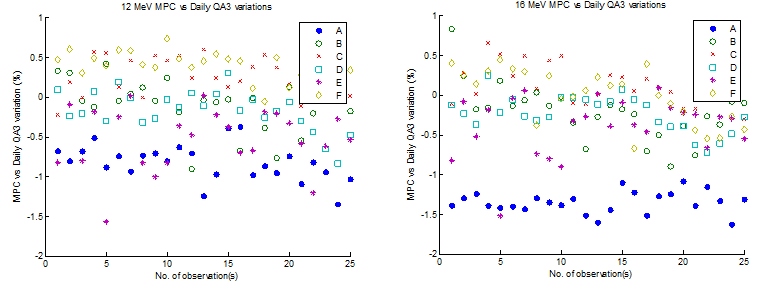


Figure 1. Individual MPC and Daily QA3 variation analysis for all machines(A-F).
